# Aortic valve chordae tendineae: A rare cause of aortic stenosis

**DOI:** 10.1016/j.radcr.2022.09.099

**Published:** 2022-10-26

**Authors:** Cara E. Saxon, Tessa S.S. Genders, Robert A. Quaife, S. James Chen, Joseph M. Burke, Erin M. McGuinn

**Affiliations:** a Department of Medicine, University of Colorado Anschutz Medical Campus, Academic Office One, 12361 E. 17th Ave, B177, Aurora, CO 80045, USA; b Division of Cardiology, University of Colorado Anschutz Medical Campus, 12631 East 17th Ave, B130, Aurora, CO 80045, USA; c Advanced Cardiac Imaging & Image Guidance Center, University of Colorado Anschutz Medical Campus, 12605 East 16th Ave, 3rd Floor, Aurora, CO 80045, USA; d Division of Cardiology, Denver Health Medical Center, 660 Bannock St Suite 5635, Denver, CO 80204, USA

**Keywords:** Aortic valve, Aortic stenosis, Chordae tendineae, Fibrous strand, TTE, transthoracic echocardiogram, LVEF, left ventricular ejection fraction, TEE, transesophageal echocardiogram, LVOT, left ventricular outflow tract, CABG, coronary artery bypass graft

## Abstract

We describe a rare case of severe low-flow, low-gradient aortic stenosis due to a calcified aortic valve chordae tendineae. The chordae was captured on cardiac computed tomography (CT) using advanced 3-dimensional image reconstruction to reveal the fibrous strand tethering the non-coronary cusp to the left ventricular outflow tract, rendering it functionally immobile. This is one of the first reported cases of severe aortic stenosis from an aortic valve chordae tendineae which highlights the utility of advanced image processing techniques in cardiac CT.

## Introduction

Aortic valve chordae tendineae have been described as rare congenital remnants of cardiac development. Previous case reports have noted fibrous strands on both bicuspid and tricuspid aortic valves, connecting aortic valve cusps to the wall of the ascending aorta, causing aortic regurgitation. Utilizing advanced cardiac computed tomography (CT), we describe the first reported case of severe low-flow low-gradient aortic stenosis due to an aortic valve chordae tethering the non-coronary cusp to the left ventricular outflow tract (LVOT).

## Case report

**Fig. 1:**
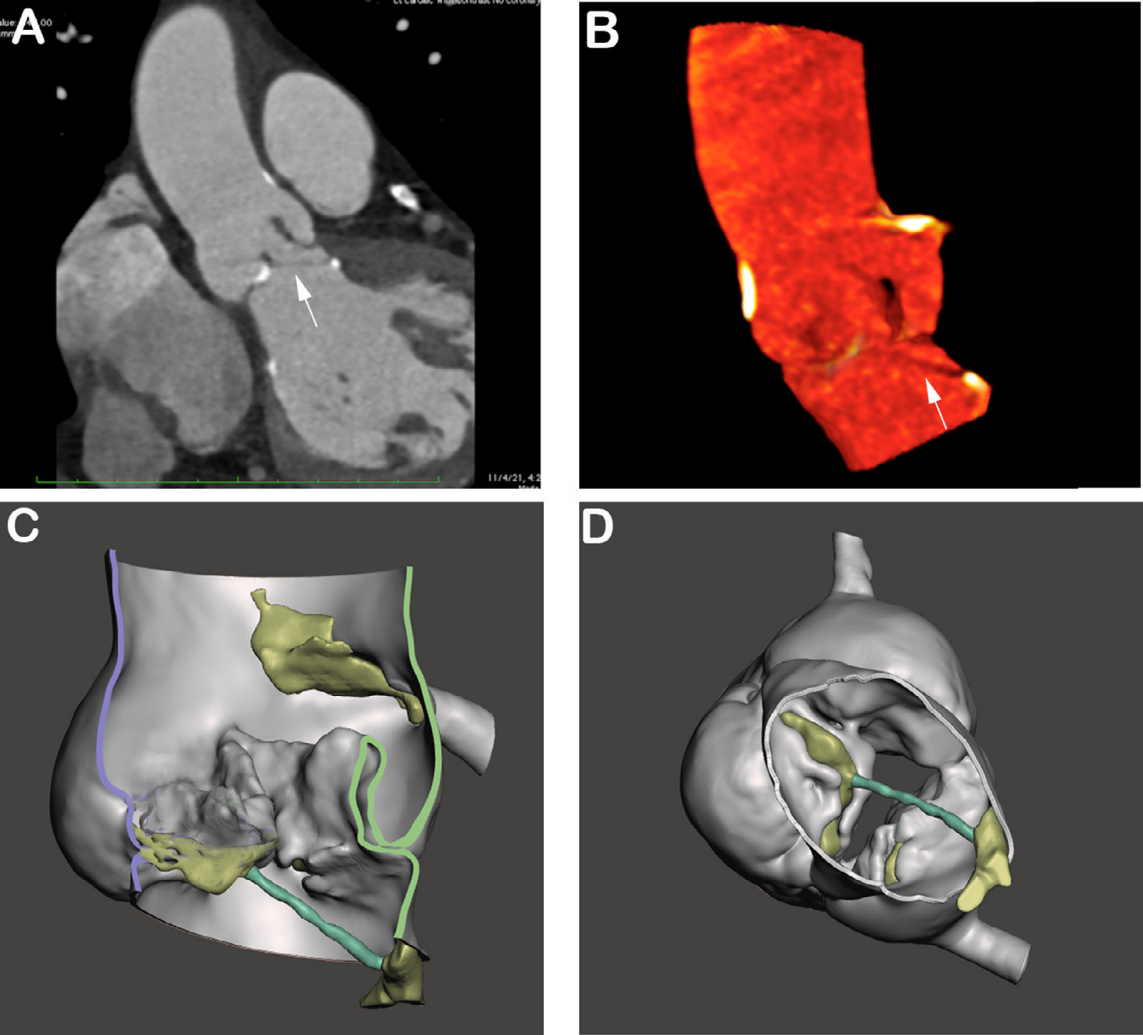
Cardiac CT of aortic valve chordae tendineae. Multi-planar reformatted image (A) and volumetric 3D display (B) of the LVOT and aortic root with a notably calcified aortic valve and fibrous strand (arrow) tethering the non-coronary cusp to left ventricular outflow tract. A right coronary cusp cut-away of a 3D STL display (C) shows a longitudinal view of chordae (teal) with aortic calcifications (yellow). A subvalvular projection of the aortic valve (D) visualizes the fibrous strand attachments to the non-coronary cusp and LVOT.

**Fig. 2:**
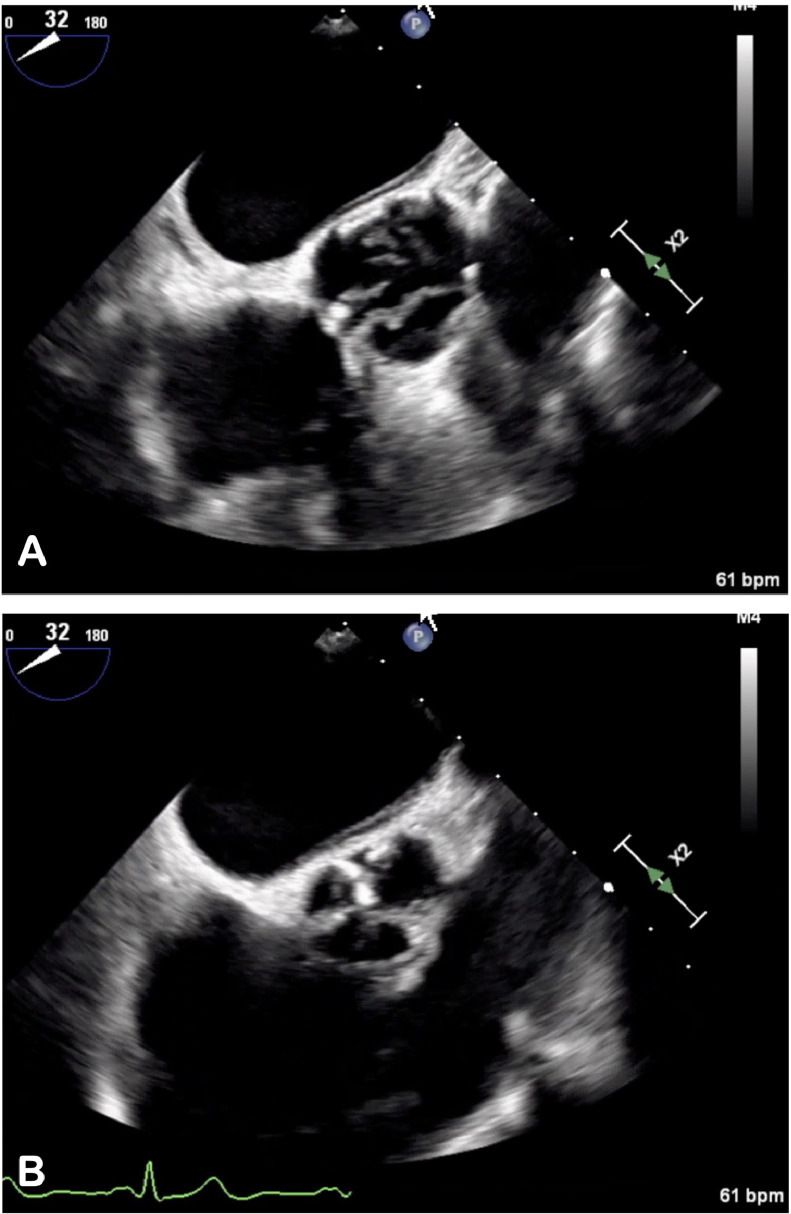
Mid esophageal TEE of aortic valve: Tricuspid heavily calcified aortic valve visualized in early systole (A) and diastole (B) on mid esophageal short axis view.

An 80-year-old female presented with one year of progressive exercise intolerance and episodes of lightheadedness upon exertion. On exam, a grade 3/6 systolic murmur was heard at the right upper sternal border. TTE revealed a LVEF of 75%, stroke volume index of 29 mL/m2, a moderately calcified aortic valve with an aortic valve area of 0.8 cm^2^, and peak velocity 3.7 m/sec. TEE demonstrated an aortic valve area of 0.94 cm^2^ by 3-D planimetry, mean gradient of 25 mmHg, peak velocity of 3.6 m/sec, and dimensionless index of 0.24, concerning for paradoxical low-flow low-gradient aortic stenosis (Supplemental Video 1). A cardiac CT was performed, and cinematic 4D display noted a fibrous strand tethering the non-coronary cusp of the tricuspid aortic valve to the LVOT, creating functional aortic stenosis (Fig. 1). Multi-planar reformatted imaging (Fig. 1A) and volumetric 3D display (Fig. 1B) of the LVOT and aortic root revealed the fibrous strand, not previously visualized on TTE or TEE (Fig. 2). She ultimately underwent a transcatheter aortic valve implantation with subsequent symptom improvement.

## Discussion

Aortic valve chordae tendineae have been described as rare remnants of embryological development. In this case, the chordal remnant was not well characterized on standard cardiac CT views (Figs. 1A & B). Discrete element segmentation of calcium and the strand clarifies the orientation and provides multiple viewpoints using stereolithography (STL) polygonal analysis (Figs. 1C & D). A right coronary cusp cut-away of a 3D STL display (Fig. 1C) shows a longitudinal view of the chordae (teal) with aortic calcifications (yellow). A subvalvular projection of the aortic valve (Fig. 1D) visualizes the fibrous strand attachments to the non-coronary cusp and LVOT.

This is one of the first reported cases of a subvalvular chordae tendineae tethering the aortic valve to the LVOT, resulting in immobilization of the associated aortic valve cusp, causing aortic valve stenosis. There are multiple case reports of fibrous strands tethering the aortic valve to the wall of the ascending aorta, causing aortic regurgitation [1], [2], [3]. One report described an incidental subvalvular aortic valve chordae noted on TEE which was surgically excised and revealed central hyalinization on pathology, consistent with a valve chordae [4]. In our case, the LVOT attachment of the aortic chordae was calcified, so it was hypothesized that calcification of the valve over time or changes in left ventricular morphology led to progressive tethering of the valve, leading to the patient's symptom development later in life.

## Patient consent

The patient provided informed consent to participate in the case report titled, “Aortic valve chordae tendineae: A rare cause of aortic stenosis” and have their data published in *Radiology Case Reports*. Only non-identifiable images were used in this submission.

## Appendix Supplementary materials

Supplemental Video 1: TEE of tricuspid aortic valve. A mid esophageal aortic valve short axis view on TEE demonstrates a tricuspid, heavily calcified aortic valve. Image, video 1
